# Valorization of Cork Using Subcritical Water

**DOI:** 10.3390/molecules25204695

**Published:** 2020-10-14

**Authors:** Mónica Cunha, Ana Lourenço, Susana Barreiros, Alexandre Paiva, Pedro Simões

**Affiliations:** LAQV-REQUIMTE, Chemistry Department, Faculdade de Ciências e Tecnologia, Universidade Nova de Lisboa, 2829-516 Caparica, Portugal; p30@fct.unl.pt (A.L.); sfb@fct.unl.pt (S.B.); abp08838@fct.unl.pt (A.P.)

**Keywords:** cork, *Quercus suber* L., subcritical water, phenolics, carbohydrates, antioxidant activity

## Abstract

Granulated cork was submitted to subcritical water extraction/hydrolysis in a semi-continuous reactor at temperatures in the range of 120–200 °C and with a constant pressure of 100 bar. The influence of temperature on the composition of the cork extracts obtained was assessed—namely, their content of carbohydrates and phenolics. The extraction yield increased with the temperature, and this was associated with the decrease in the dielectric constant of water and the increase in its ionic product. Extracts composed of up to 36% phenolics were obtained at temperatures of up to 120 °C, with an antioxidant activity only two times lower than that of pure gallic acid, but in low amounts. Assays at higher temperatures generated extracts richer in carbohydrates and with a phenolics content of ca. 20 wt.% in comparatively far higher amounts. Neither the amount of suberin nor its structure were affected by the subcritical water treatment.

## 1. Introduction

Cork is a thick, continuous layer that envelops the stems of certain species of oak trees in Southern Europe and Northern Africa, serving as a protective layer between the tree’s living cells and the environment. As a natural and renewable material harvested from the bark of *Quercus suber* L., cork has considerable economic value [1]. Portugal is the world leader in the cork sector, having exported 201 thousand tons in 2018 (more than 90% of its production), which represents a share of 62.5% of the world market [2]. Some the unique characteristics of cork are its low density, low permeability to both gases and liquids, elastic compression and recovery, low thermal and electrical conductivity, acoustic insulation, resistance to abrasion, fire retardant qualities, and hypoallergenic nature. These properties have made cork an attractive material for a wide range of sectors, from building construction to aeronautics. However, about 70% of all the cork used finds application in the wine industry in the form of cork stoppers [2].

The main by-products of the cork industry are cork granulates that result from the transformation of raw cork into cork stoppers. Cork granulates consist mainly of scraps and parings from the cutting stage, together with the material rejected at the selection stage of natural cork stoppers. Granulates of differing granulometry are first agglutinated, and then subjected to heat and pressure in autoclaves to form expanded agglomerates, if they are agglutinated without the use of any synthetic agents, or to form composite agglomerates if synthetic resins are used [3]. 

Both the manufacturing of cork stoppers and the formation of cork agglomerates produce waste, essentially rejected material that represents 20–30% of the industry’s raw cork feed [4]. Cork powder or dust, which makes up most of the cork waste, is usually burned to produce energy, while the remaining rejected granulate is reused in the manufacturing process. Though mostly recycled in some form, cork waste remains a by-product of the cork industry with a low commercial value [4,5].

Cork consists mostly of suberin (ca. 40%), lignin (ca. 22%), carbohydrates (ca. 18%), extractives (ca. 15%), and inorganics (ca. 1%) [6]. There has been growing interest in the extractives present in cork and cork by-products, more specifically in the bioactive compounds, such as polyphenols and triterpenoids (e.g., friedelin), for their anti-inflammatory, anti-cancer, anti-oxidative, anti-viral, anti-fungal, and anti-bacterial properties [7].

The polyphenols found in cork and cork by-products have seen an increase in applications in the food, pharmaceutical, and cosmetics industries [5]. The phenolic compounds from cork and cork by-products are most often obtained through solvent extraction. One common method involves the use of methanol/water mixtures, sometimes followed by extraction with an organic solvent, such as ethyl ether [8]. Another process involves sequential extraction with solvents of increasing polarity (dichloromethane, methanol, and water) to fractionate the cork extracts into separate lipophilic and phenolic fractions [9]. A comparative study that used a variation of both of these methods, with the initial removal of the lipophilic fraction from all samples, reported the generation of extracts with 20 to 35 g of phenolics/100 g extract, corresponding to a recovery of about 2.4 to 10.6 mg of phenolics/g cork [10]. Bouras et al. [11] employed microwave-assisted extraction to obtain extracts from *Quercus* bark using mixtures of three different solvents—namely, methanol, ethanol, and water—in different proportions. The authors reported a total phenolic content of the extracts ranging from 1.6 to 2.1 g of phenolics/100 g extract. Recently, a process for extracting phenolic compounds from cork granulates using a water/propylene glycol mixture has been described in a patent application. Seven different phenolic compounds were identified in the hydro-glycolic extracts, corresponding to a recovery of 10 to 16 mg of phenolics/g of cork granulate [12]. 

Cellulose, hemicellulose, and lignin, the three major lignocellulosic components of the plant cell walls of cork, are also important natural bioresources, not only in producing biofuels, but also in obtaining several value-added chemicals. However, most of the chemical processes used to decompose and extract these components involve the use of acid, alkali, and organic solvents [13]. A green alternative for the processing of biomass is the use of pressurized hot water, or subcritical water (SBW). SBW is liquid water at high temperatures and above its vapor pressure. At these conditions, the dielectric constant of the water decreases and its ionic product increases, making water a more reactive medium for the hydrolysis of lignocellulosic matrices [14]. 

The present work focuses on obtaining extracts from granulated cork of the *Quercus suber* L. species, enriched in different value-added compounds, using a semi-continuous, SBW treatment.

## 2. Results and Discussion

### 2.1. Chemical Characterization of Granulated Cork

The water content of the original granulated cork was 5.6 ± 0.2 g/100 g of cork. The main component, as shown in Table 1, was suberin, at ca. 41 g/100 g of dry granulated cork. This is within the range reported by other authors [15,16,17], although the suberin content has been shown to vary significantly in raw unprocessed cork samples, even those collected from the same tree [1,15,16]. After suberin, the more predominant components of cork are lignin and carbohydrates, with suberin:lignin and lignin:carbohydrates ratios falling within the range of values reported in the literature for virgin cork from *Quercus suber* L. (the main oak species in Portugal) [15]. Cellulose, a homopolysaccharide composed of glucose monomers, accounts for slightly less than half of the total amount of carbohydrates of the granulated cork, the rest being the heteropolysaccharide hemicellulose, made of the sugar monomers, similar to what has been observed by other authors [15]. An HPLC chromatogram of the monosaccharides of the granulated cork is given in Supplementary Figure S5.

Granulated cork has a significant content of extractives, up to 11.4 wt.% of dry matter, although a little lower than virgin cork, with 15.3 wt.% on average [15]. Non-polar substances such as triglycerides, waxes, and triterpenes, soluble in *n*-hexane, account for approximately a third of the total extractives. Polar compounds, including, among others, polyphenols and soluble sugars, extracted by ethanol and water, make up the rest of the cork extractives. The total phenolic content (TPC) of the combined ethanol and water extracts, measured by the Folin–Ciocalteau colorimetric method, was found to be 3.9 ± 0.3 g GAE/100 g dry cork granulate. A previous study on the phenolic content of raw cork extractives reports values of up to 1.99 ± 0.01 g GAE/100 g dry cork [18]. The total carbohydrates content (TCC) of the combined ethanol and water extracts was found to be 1.41 ± 0.07 g GE/100 g dry cork granulate, a relatively low amount when compared to other lignocellulosic biomass matrices [19,20,21]. Other minor components—namely, ash and protein—fell within the ranges observed in the literature for virgin cork [9].

### 2.2. Efficiency of SBW Extraction/Hydrolysis

The influence of temperature and water flow rate on the yield of the SBW extraction/hydrolysis of granulated cork can be seen in Table 2.

Temperature had a strong effect on all the parameters monitored. This can be attributed to the increase in the ionic product of water with increasing temperature. In the temperature range 150–200 °C, the ionic product of water is about three orders of magnitude higher than at room temperature (an increase from p K_w_ = 14 to ca. 11 [22]), thus promoting the hydrolysis of biomass components—namely, structural carbohydrates. In addition, temperature affects the kinetics of the hydrolysis reaction itself. Increasing the temperature increases the thermal energy, thereby increasing the reaction rates of hydrolysis [23].

The highest recovery of carbohydrates occurred at 200 °C, with an overall yield of 7.27 g of carbohydrates/100 g of granulated cork, which corresponds to roughly 40% of the total content of carbohydrates available in granulated cork. At 120 °C, SBW should only be able to access soluble sugars. At 150 °C, the results in Table 2 suggest that SBW was able to remove a fraction of hemicellulose. At 200 °C, the results suggest that ca. 70% of the hemicellulose was removed as well by SBW, with the resilience of cellulose preventing its depolymerization by SBW at that temperature.

The extraction yield of phenolics followed a similar pattern: the higher the temperature reached in the assay, the higher the yield of phenolics obtained, reaching 3.76 g/100 g granulated cork at 200 °C. This amount corresponds to ca. 96% of the total content of phenolics available in the original raw cork. The dielectric constant of water decreases sharply with increasing temperature, varying from 78.5 at 25 °C to ca. 35 at 200 °C, a value that is comparable to the dielectric constant of methanol at room temperature and pressure [24]. At such conditions, subcritical water gains the ability to dissolve less polar compounds.

Table 2 gives the overall yields. As indicated in the section on semi-continuous SBW extraction, the liquors produced in each assay were divided into separate samples. Each sample corresponded to all the liquor collected for a given temperature interval, as the water outlet temperature rose from ambient to 50 °C, from 50 °C to a higher temperature, and so forth. The analysis of each of these samples enables a more detailed analysis of the effect of temperature on the SBW treatment, as shown in Table 3.

While the highest recovery of phenolics was achieved in the 200 °C assay, the extract with the highest phenolic content was obtained in the assay targeting 120 °C as temperature increased from 50 to 120 °C. In fact, 1.3 g of this extract had 0.47 g of phenolics, which corresponds to ca. 36 wt.%. However, the amount of extract obtained at such conditions is very small. In the assay targeting 200 °C, for instance, as temperature increased from 120 to 200 °C, 11.6 g of extract was produced with a TPC of ca. 22 wt.%, while, when the temperature was kept constant at 200 °C, 4.2 g of extract was produced, with a TPC of ca. 22.6 wt.%.

The results of the HPLC analysis of the phenolic compounds in the SBW extracts are shown in Table 4 for the assay targeting 200 °C. HPLC chromatograms are shown in Supplementary Figures S1–S4. Gallic acid was found to be the phenolic compound present in the highest concentration. Caffeic acid and ferulic acid were present in relatively lower amounts. Ellagic acid was only found in the extracts obtained at temperatures higher than 120 °C. This phenolic acid is known to have antioxidant and other beneficial properties, although it shows a poor water solubility and low bioavailability. It is understood that its planar and symmetrical structure, associated with irreversible bindings to cellular DNA and proteins, is the cause of the very poor bioavailability of ellagic acid [25]. It is possible that SBW is only able to extract ellagic acid at higher temperatures, since at such conditions the ionic product of water makes it a more reactive medium for hydrolysis. Furthermore, at 200 °C the dielectric constant of water is similar to that of methanol at room temperature, as mentioned earlier (ellagic acid is fairly soluble in methanol).

The values obtained in this work agree with those reported in the literature, considering the natural variability of cork composition with the collection year and site, as well as the type of raw material used. Mislata et al. [26] obtained ethyl acetate extracts from granulated corks macerated in a hydroalcoholic solution. They identified gallic acid as the phenolic species with highest concentration in the extracts, with values of between 60.6 and 180.9 μg/g_extract_, followed by protocatechuic acid, varying between 41.3 and 161.6 μg/g_extract_. Batista et al. [12] obtained a water/propylene glycol extract from granulated cork. The gallic acid and protocatechuic acid contents of their extracts, 60–100 μg/g cork and 100–130 μg/g cork, respectively, are within the range of our values. On the other hand, ellagic acid (6800–8200 μg/g cork) and castalagin (1800–2100 μg/g cork) were identified in higher concentrations than our SBW extracts. The same was reported by Santos et al. [10] for a methanol extract obtained from cork powder. The major phenolic compounds identified were ellagic acid, followed by gallic, protocatechuic, and caffeic acids and esculetin.

### 2.3. Antioxidant Activity

The antioxidant activity of each of the samples collected at various temperature ranges, expressed here as the half maximal effective concentration, or EC_50_, is shown in Table 5.

For comparison, pure gallic acid analyzed under the same conditions showed an EC_50_ of 0.035 ± 0.001 mg/mg DPPH.

The lowest value of EC_50_ obtained—at 0.25 mg extract/mg DPPH—and therefore the highest antioxidant activity was obtained for the extract collected in the temperature range between 50 °C and 120 °C. This range of temperatures generally corresponded to the higher TPC in all the assays performed. Taking the TPC of the 50–120 °C extract into account, the earlier value would correspond to an EC_50_ of ca. 0.07 mg phenolics/mg DPPH, only two times higher than the EC_50_ of the gallic acid standard. At higher temperatures, the slight decrease in the TPC of the extracts was accompanied by a decrease in the antioxidant activity, with the EC_50_ varying from 0.46 to 0.51 mg extract/mg DPPH in the 120 °C to 200 °C temperature range. Increasing the temperature led to higher concentration of carbohydrates in the extracts, and this may explain the decrease in the antioxidant activity, if compared with the samples obtained at lower temperatures. Furthermore, ellagic acid was only found in the latter extracts. The EC_50_ of pure ellagic acid under the same conditions of analysis led to a value of 0.047 ± 0.001 mg/mg DPPH, which is slightly higher than that for pure gallic acid. This may also partly explain the decrease observed in the antioxidant activity of the 120–200 °C extracts when compared with the others.

Even so, the EC_50_ values obtained for cork extracts are similar or even better than those obtained previously for extracts from white wine grape pomace (0.53 to 1.8 mg extract/mg DPPH) [19] and spent coffee grounds (0.6 to 3 mg extract/mg DPPH) [27]. For comparison, a water infusion of as-received granulated cork at normal boiling temperature was carried out for one hour, using a volume of solvent to cork mass of ca. 190 mL/g. The extraction yield obtained was 2.4 g/100 g cork. The extract had a TPC of 55 g/100 g of extract and an EC_50_ of 0.37 mg extract/mg DPPH. Although this extract had a higher phenolic content than the SBW extracts, its antioxidant activity was lower, possibly explained by other extractives solubilized by “normal” hot water.

The EC_50_ values obtained for the SBW extracts of granulated cork compare well with those reported in the literature for cork extracts. Aroso et al. [28] extracted cork powder with different solvents, water, ethanol, and respective mixtures, and the respective EC_50_ values ranged from 7.9 to 13 μg extract/mL (for comparison, our results varied between 10 to 19 μg extract/mL). Santos et al. [10] reported an EC_50_ of 3.6 to 5.8 μg/mL for the methanol/water extracts from cork powder.

The SBW extracts of granulated cork exhibited a high antioxidant activity, showing potential for applications in the cosmetics, food, and pharmaceutical industries.

### 2.4. Suberin and Lignin Extraction

Due to the low amounts of solid extract collected in each SBW extraction run (less than 2 g of extract in total), the suberin and lignin extraction yield was determined indirectly through the difference between their content in the original cork and in the residue of the SBW assay at 200 °C.

The SBW residue showed a lignin and carbohydrate content of 17.8 g/100 g dry cork and 12.0 g/100 g dry cork, respectively. Compared with the respective content in the original granulated cork, it is possible to conclude that the subcritical water extracted ca. 29% of the lignin initially present in the cork, and ca. 35% of the total amount of carbohydrates in the granulated cork. The latter is relatively close to the total amount of carbohydrates quantified in the SBW extracts.

The quantification of suberin content in both the original granulated cork and the residue of the SBW extraction assay at 200 °C gave similar values within the experimental error. This may indicate that subcritical water at the range of temperatures studied in this work did not significantly hydrolyze the cork suberin. Nevertheless, the suberin monomers present in both the original cork and the SBW residue were analyzed through FTIR and NMR spectroscopy to assess if the SBW was still able to alter the structure of suberin during the assay.

**Figure 1:**
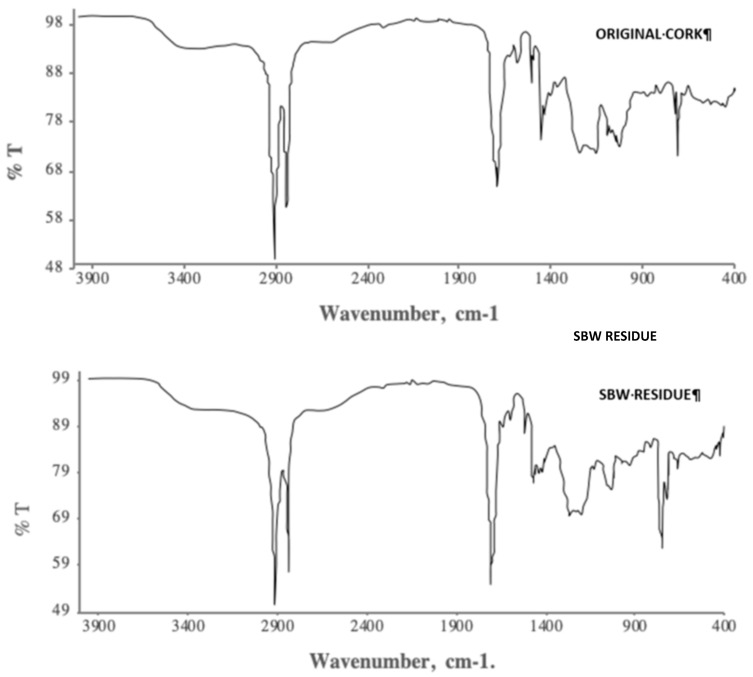
FTIR spectrum of the isolated suberin monomers from granulated cork, before and after the SBW treatment.

**Figure 2:**
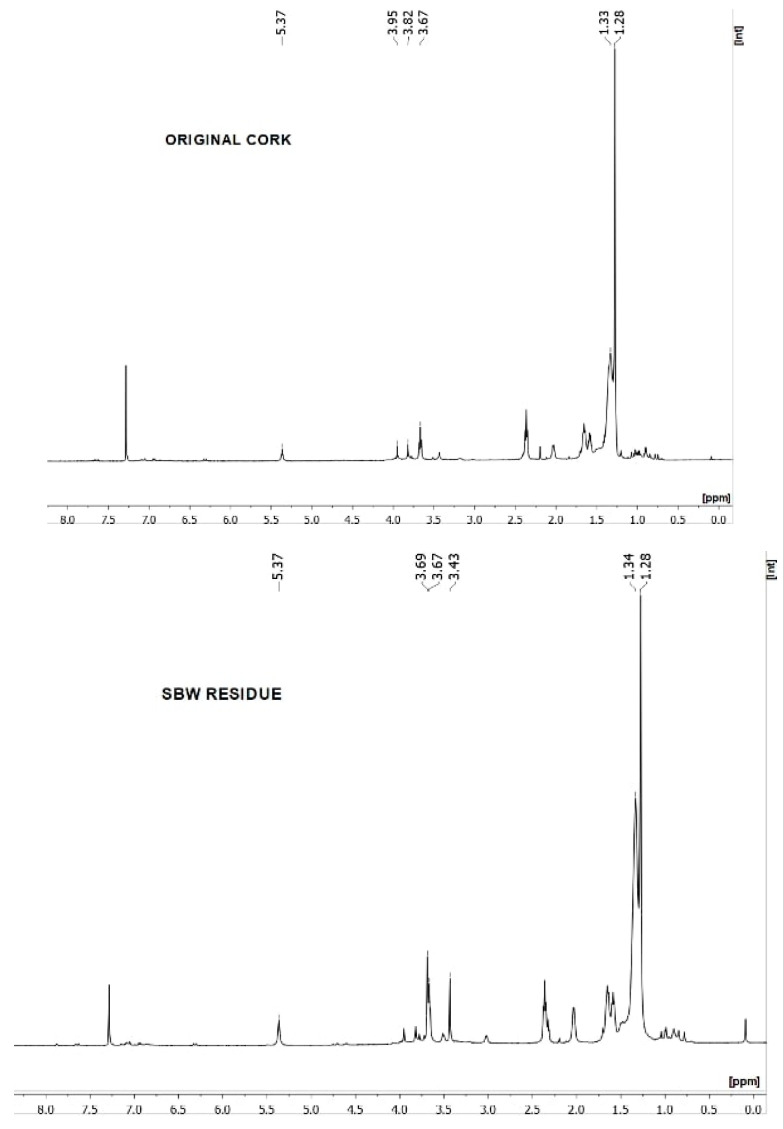
^1^H NMR spectra of the suberin monomers of granulated cork, before and after the SBW treatment, in deuterated chloroform (CDCl_3_).

The FTIR spectrum and the solid-state ^1^H-NMR spectrum of the isolated suberin monomers of the original granulated cork and of the SBW cork residue are shown in Figure 1 and Figure 2.

The FTIR spectrum of our granulated cork is comparable to the one shown for an extractives-free pure cork sample [1]. The broad band with a maximum at 3300 cm^−1^ can be attributed to hydrogen-bonded O–H groups (intermolecular). The aliphatic nature of the suberin structure is characterized by strong absorptions at 2912, 2849, and 1473 cm^−1^ (methylene, –CH_2_–). Strong bands at 1707 cm^−1^ (carbonyl, C=O) and 1257 cm^−1^ (alkyl-aryl ethers, =C–O–C) also indicate the prevalence of the ester moieties that typically characterize the ester linkages in the suberin structure. The band at 1600 cm^−1^ can be attributed to C=C double bonds [29].

In the ^1^H-NMR spectrum, the signal at δ 5.3 ppm corresponds to alkenyl protons (CH_2_=CH–). The signals in the region between δ 3.9 and δ 3.4 ppm can be attributed to methylene protons vicinal to oxygen atoms of the ester functions and to the methyne of the glycerol moiety. The signal at δ 3.4 ppm was assigned to the methoxy groups (–O–CH_3_) that resulted from the methanolysis. Signals that could be attributed to the aromatic moieties in the suberin structure are negligible or entirely absent in this spectrum. The alkaline methanolysis method used in this study favors the removal of the aliphatic chains that make up the majority of suberin’s aliphatic domain, which might explain not only these negligible aromatic signals but also the predominance of the signals in the aliphatic regions (δ 1.3 and δ 1.2 ppm) of both spectra. The same can be said about the absence of peaks assigned to carboxyl moieties (–COOH) [29].

Comparing the FTIR and ^1^H-NMR spectra of the original granulated cork and the SBW residue, one cannot observe significant differences between them, thus indicating that the structure of suberin was not altered during the SBW hydrolysis of granulated cork.

## 3. Materials and Methods

### 3.1. Materials

The granulated cork (0.5–1 mm particle size) kindly provided by a Corticeira Amorim (Mozelos, Portugal), was stored in plastic bags at room temperature.

All the reagents used in this work were of high grade. Chloroform (99%) was from Carlo Erba Reagents (Barcelona, Spain), methanol (99%) phenol, and sulfuric acid (96%) were from Sigma-Aldrich Co. (St. Louis, MO, USA), and ellagic acid, gallic acid, caffeic acid, and ferulic acid were from Merck KGaA (Darmstadt, Germany).

### 3.2. Chemical Characterization of Granulated Cork

The water content of granulated cork was measured in a thermogravimetric balance (Kern DAB 100-3) at 105 °C.

The ash content was determined gravimetrically, through weight difference, after placing a porcelain crucible containing ca. 0.3 g of sample in a muffle at 550 °C for 6 h and then in a desiccator to cool down.

The protein content was determined indirectly by the measuring nitrogen content through elementary microanalysis, using a nitrogen-to-protein conversion factor of 6.25 [30].

A series of extractions were performed with solvents of increasing polarity to remove all the extractives in a sequence of three steps [1]. The first step was a Soxhlet extraction with 70 mL of *n*-hexane (at 69 °C) for 3 h to remove non-polar compounds, mostly triterpenes, long chain alkanes, and alkanols. The second step was a Soxhlet extraction with 70 mL of ethanol (at 78 °C) for 3 h, and the third step consisted of an infusion using 100 mL of water, previously heated to normal boiling temperature, for 1 h. Ethanol and water were used to remove polar compounds, mostly phenolics, such as tannins and flavonoids, as well as soluble carbohydrates, which make up the remaining cork extractives [1,9]. Each extract was filtered, the solvent evaporated, and the remaining solid weighed to quantify extractives. The final residue was dried overnight at 45 °C to remove the remaining solvent before weighing.

The total phenolic content and the soluble carbohydrate content of the ethanol Soxhlet extracts and the extracts obtained by infusion in water were determined by the Folin–Ciocalteu and the phenol-sulfuric acid method, respectively, as described in a further section.

The extractives-free granulated cork was submitted to an alkaline methanolysis reaction to extract and isolate suberin [9,29,31]. The complete depolymerization of suberin was achieved by treating, overnight, 150 mg of the sample with 30 mL of a refluxing mixture of a freshly prepared, dry, 1.0 M solution of sodium methoxide (NaOMe), a common reagent used to induce the ester cleavage of the suberin structure. The reaction mixture was then filtered. The solid obtained was washed with methanol until a clear liquid was obtained. This liquid was added to the supernatant, yielding a solution that was neutralized to pH of 5–6 by adding small quantities of a 2.0 M solution of sulfuric acid in methanol. The solvent was evaporated and the residue was suspended in 75 mL of water. The suberin monomers in this solid were isolated by extraction with 2 × 75 mL of chloroform. The solution obtained was dried with anhydrous sulphate, filtered, evaporated to dryness, and dried under a vacuum, yielding a paste-like material composed of fatty aliphatic monomers of suberin that were quantified by weighing.

Suberin monomer analysis was carried out by Attenuated Total Reflection Fourier Transform Infrared (FT-IR) spectroscopy, using a Perkin Elmer Spectrum 1000 spectrophotometer (PerkinElmer, Waltham, MA, USA), and by ^1^H-NMR (Nuclear Magnetic Resonance) spectroscopy, using a Bruker AVANCE III 400 apparatus (Bruker, Billerica, MA, USA), after dissolving the suberin monomers in deuterated chloroform.

The solid obtained after the sodium methoxide treatment—suberin-free material—was further washed with water, dried in an oven at 45 °C, and stored for further analysis. The insoluble structural carbohydrates in extractives-free and suberin-free material were quantified through a two-step concentrated acid hydrolysis. To that end, the suberin-free sample was treated with a 72% (*w*/*w*) sulfuric acid solution in a 30 °C water bath for 1 h, after which the mixture was diluted with 84 mL of water to a 4% (*w*/*w*) concentration, before being incubated in a silicone bath at 121 °C for 1 h. The resulting mixture was filtered and the monosaccharide content of the supernatant was determined by HPLC analysis, as described in a further section. The acid soluble lignin (ASL) of the supernatant was determined through the direct reading of its absorbance in the UV spectrum (GE Healthcare, Chicago, IL, USA), at 205 nm. First, the supernatant was diluted with water until its absorbance fell between 0.7 and 1.0. The ASL was calculated using the following equation:

(1) $$\%\;ASL\;=\;\frac{UV_{abs}\;\times \;Volume_{filtrate\;}\;\times \;DF}{\varepsilon xODW_{sample}\;\times \;Pathlength}\;\times \;100.$$

The residue from the acid hydrolysis was washed, dried, and weighed, and the acid insoluble (Klason) lignin content was calculated after taking into account the protein and acid insoluble ash from the weight of this dry residue [32].

The suberin content of the residue that remained in the reactor after the SBW extraction was determined using the protocol applied for the original cork granulate.

### 3.3. Semi-Continuous SBW Extraction

**Figure 3:**
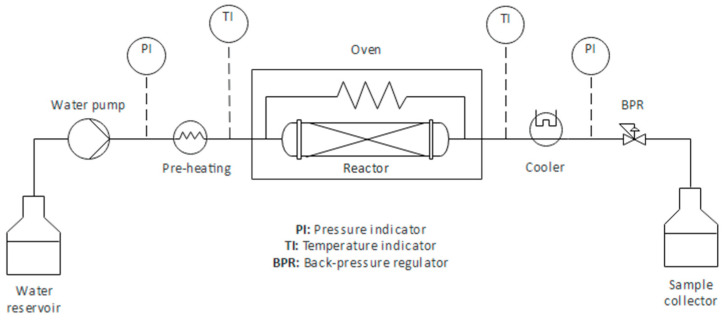
SBW semi-continuous unit for the extraction/hydrolysis of granulated cork.

The extraction of granulated cork was performed in an SBW unit shown schematically in Figure 3. In each experiment, distilled water was pumped using a high-pressure pump (KNAUER Preparative Pump model 1800) (Knauer, Berlin, Germany). The water filled the reactor, a 51 cm-long and 2.6 cm-internal-diameter-wide stainless steel tube, which was previously loaded with ca. 10 g of granulated cork as received, and placed inside an electric oven with temperature control (Nabertherm model 30–3000 °C N641) (Nabertherm GmbH, Lilienthal, Germany); the pressure was allowed to reach 100 bar, as controlled by a back-pressure regulator valve (Tescom model 26–1700) (Tescom Europe GmbH & Co. KG, Selmsdorf, Germany). To prevent the thermal degradation of the raw material, it is only at this point that both the heating cord around the inlet water piping, connected to a temperature controller, and the heating program of the oven where the reactor is placed were activated. This marked the start of the process (t = 0), and from this moment onward the liquor leaving the reactor was continuously collected for analysis. The outlet water stream passed through a 15 µm filter before being depressurized, cooled down in an ice bath, and collected for analysis. The pressure and temperature of both the inlet and outlet streams were monitored throughout the experiments. The pressure was kept at 100 bar throughout all the experiments to ensure that the water was always a liquid. The SBW water flowrate was fixed at 10 mL/min for all the assays. The target temperatures were 120, 150, and 200 °C.

The influence of temperature on the SBW extraction was determined by separating into different sampling tubes the amount of liquor collected. For example, in the case of the 200 °C assay, sample 1 was collected from the t = 0 to the time at which the temperature of the outlet stream reached 50 °C. Sample 2 was collected between an outlet temperature of 50 and 120 °C. Sample 3 was collected between 120 and 200 °C. Lastly, sample 4 was collected at an outlet temperature of 200 °C for a duration of 30 min.

The extraction yield was calculated by measuring the total amount of liquor collected in each sample, determining the amount of extract obtained by lyophilizing a given volume of liquor from that sample, correcting for the total volume of the sample, and finally summing up for all four samples [27].

At the end of the extraction, the heating was turned off and the system was allowed to cool down. Known amounts of each of the liquors collected were stored at 4 °C, to be used for the subsequent quantification of the phenolic and carbohydrate content. The residue that remained in the reactor after the SBW extraction was washed with water and dried in an oven at 100 °C overnight.

### 3.4. Phenolic Analysis

The total phenolic content (TPC) of the SBW liquors, as well as the ethanol Soxhlet and the water infusion extracts, was determined through the Folin–Ciocalteau colorimetric method [33]. To that end, a gallic acid solution was used to build a calibration curve. Due to the possible interference of protein with phenolics quantification, a step of protein precipitation was performed [34]. This consisted of adding 120 µL of 100% (*w*/*v*) trichloroacetic acid to 800 µL of sample. The mixture was stirred and stored at −20 °C for 5 min, then at 4 °C for 15 min, after which it was centrifuged (12,000× *g*, 15 min) and the protein precipitate was discarded. To 20 µL of both the recovered supernatant and the standard gallic solutions were added 1.58 mL of distilled water and 100 mL of Folin–Ciocalteu reagent. The mixtures were stirred and incubated at room temperature for about 5 min, after which 300 µL of sodium carbonate solution were added, followed by incubation in a dry bath at 40 °C for 30 min. The absorbance was measured at 750 nm, and the calibration curve was used to calculate the TPC, expressed here as grams of gallic acid equivalents per liter (g_GAE_/L).

The individual phenolic compounds of SBW granulated cork extracts were identified through HPLC analysis. The analysis, which was performed using a method adapted from the literature [35], was performed with an Agilent Infinity 1100 system, using an injection volume of 20 µL and a flow rate of 0.3 mL/min. The column (Waters NOVAPAC C18 150 × 3.9 mm, 4 µm pore size) (Waters Corporation, Milford, MA, USA) was kept at a constant temperature of 25 °C. The absorbance was measured at 280 nm using a diode array detector. The mobile phase was a mixture of solvents, (A) water/acetic acid (99:1; *v*/*v*) and (B) water/acetonitrile/acetic acid (79:20:1; *v*/*v*/*v*), with the gradient 80–20% A for 55 min, 20–10% A from 55 to 70 min, and 10–0% A from 70 to 90 min. The chromatographic column was washed with 100% B for 10 min and then stabilized at the initial conditions for another 10 min.

The standards of gallic acid, caffeic acid, ferulic acid, and ellagic acid were prepared and analyzed for identification purposes.

### 3.5. Carbohydrate Analysis

The amount of sugars was determined through HPLC analysis, with a method adapted from the literature [36]. The analysis was performed using a Dionex ICS-3000 system, with electrochemical detection, using a 4 × 50 mm Thermo BioLC Dionex AminoTrap precolumn and a 4 × 250 mm Thermo Dionex CarboPac SA10 column, with an injection volume of 10 µL and a constant temperature of 25 °C. A 1 mM NaOH solution was used as a mobile phase, at a constant flow rate of 1 mL/min. Calibration curves were built for the monosaccharides (concentrations between 5–100 mg/L).

The total carbohydrate content (TCC) in the SBW liquors was measured through the phenol-sulfuric acid method. For this method, D(+)-glucose monohydrate solutions were used to build a calibration curve. To 500 µL of sample were added 1.5 mL of sulfuric acid (96%) and 300 µL of a 5% phenol aqueous solution. The mixtures were stirred and incubated at 90 °C in a dry bath for 5 min, after which the mixtures were stirred once more and cooled down to room temperature in a water bath. Absorbance was measured at 490 nm, and the calibration curve used to calculate the TCC (expressed here as g/L glucose equivalent -GE).

The methods referred above were also used to analyze the solutions obtained during biomass characterization.

### 3.6. Antioxidant Activity

The antioxidant activity of each SBW extract, as well as the ethanol Soxhlet and water infusion extracts, was determined through the 2,2-diphenyl-1-picrylhydrazyl (DPPH) assay [37].

To that end, a stock solution of 24 mg of DPPH in 100 mL of methanol was prepared and stored at −20 °C for at least 2 h. A small amount of this solution was then diluted by adding methanol, until its absorbance (measured at 517 nm) reached near 1. In each DPPH assay, to 4 mL of this final adjusted solution were added 150 µL of solutions of extract in (50:50, *v*/*v*) H_2_O:EtOH (range of concentrations between 50 and 2000 mg/L) or 150 µL of only H_2_O:EtOH, with no extract, in the case of the blank.

The mixtures were stirred and stored in the dark for 40 min at room temperature, after which their absorbance was measured (at 517 nm). To determine the samples’ antioxidant activity, the scavenging of the free radical was first calculated using the following equation:

(2) $$\%\;Inhibition\;=\;\frac{A_{DPPH}-A_{sample}}{A_{DPPH}}$$

where *A_DPPH_* is the absorbance of the blank and *A_sample_* is the absorbance of the sample with the extract. The antioxidant activity of the extracts is then measured through the half maximum effective concentration (EC_50_), which is calculated from the inhibition curves obtained [19].

## 4. Conclusions

Granulated cork was submitted to subcritical water extraction/hydrolysis in a semi-continuous reactor at different conditions of temperature, with the goal of obtaining extracts enriched in different value-added compounds. Carbohydrates-rich extracts were obtained at temperatures in the 120–200 °C range. Ca. 70% of the cork hemicellulose was extracted, with cellulose remaining in the original material. SBW extracted ca. 96% of the total content of phenolics available in the granulated cork. Phenolics-rich extracts, with a content of up to 36 g of phenolics/100 g of extract, were obtained at the lower temperature range of 50–120 °C. All the SBW extracts exhibited a high antioxidant activity, with that of the extract collected between 50 and 120 °C being only two times lower than the antioxidant activity of gallic acid, showing potential for applications in the cosmetics, food, and pharmaceuticals industries. On the other hand, suberin was not extracted by SBW, and its structure remained intact in the granulated cork, which can thus be used like the original material in many sectors.

## Figures and Tables

**Table 1:** Composition of granulated cork on a dry weight basis.

Component	wt.%
Ashes	0.89 ± 0.02
Extractives	11.4 ± 2.2
*n*-Hexane	3.3 ± 0.4
Ethanol	4.4 ± 0.8
Water	3.7 ± 1.0
Suberin	41.0 ± 3.4
Lignin	24.9 ± 3.4
Soluble	1.6 ± 0.2
Insoluble	23.3 ± 3.2
Carbohydrates	18.4 ± 5.2
Cellulose ^1^	7.8 ± 2.1
Hemicellulose	10.6 ± 3.1
Protein	3.2 ± 0.1

^1^ Measured as glucose.

**Table 2:** Effect of temperature on the SBW extraction/hydrolysis of granulated cork.

Temperature (°C)	Extraction Yield (g/100 g Cork)	Yield of Carbohydrates (g/100 g Cork)	Yield of Phenolics (g/100 g Cork)
120	2.17 ± 0.07	0.59 ± 0.02	0.73 ± 0.04
150	10.5 ± 0.3	2.86 ± 0.16	2.15 ± 0.10
200	17.0 ± 0.5	7.27 ± 0.06	3.76 ± 0.18

**Table 3:** Cumulative extraction yields of the SBW assays with granulated cork.

Target Temperature (°C)	Temperature of Sample Collection (°C)	Extraction Yield (g/100 g Cork)	Yield of Carbohydrates (g/100 g Cork)	Yield of Phenolics (g/100 g Cork)
120 °C	<50	0.3	0.07	0.07
	50–120	1.6	0.44	0.54
	120	2.2	0.59	0.73
150 °C	<50	0.7	0.09	0.17
	50–150	7.3	1.43	1.61
	150	10.5	2.86	2.15
200 °C	<50	0.3	0.09	0.08
	50–120	1.2	0.25	0.31
	120–200	12.8	6.49	2.81
	200	17.0	7.27	3.76

**Table 4:** Phenolic compounds in the SBW extracts obtained in the assay targeting 200 °C.

T (°C)	Phenolic Content (mg/g_extract_)					Phenolic Content (μg/g_dry cork_)		
	Ellagic Acid	Gallic Acid	Caffeic Acid	Ferulic Acid	Ellagic Acid	Gallic Acid	Caffeic Acid	Ferulic Acid
<50		4.7 ± 0.8	0.6 ± 0.1	0.8 ± 0.1		14.9 ± 2.6	2.0 ± 0.4	2.6 ± 0.5
50–120		4.9 ± 0.9	0.5 ± 0.1	0.6 ± 0.1		61.2 ± 10.9	6.5 ± 1.1	7.5 ± 1.4
120–200	1.4 ± 0.2	4.5 ± 0.8			147.2 ± 26.2	524.6 ± 93.4		
200	2.1 ± 0.4	3.0 ± 0.5	0.13 ± 0.02	0.4 ± 0.1	86.8 ± 15.5	123.9 ± 22.1	5.5 ± 0.9	14.8 ± 2.7

**Table 5:** Phenolic compounds in the SBW extracts obtained in the assay targeting 200 °C.

Temperature (°C)	EC_50_ (mg extract/mg DPPH)	TPC (g/100 g extract)
<50	0.406 ± 0.007	26.7 ± 2.1
50–120	0.253 ± 0.001	25.6 ± 0.2
120–200	0.459 ± 0.006	21.6 ± 1.3
200	0.510 ± 0.004	22.6 ± 0.4

## Supplementary Materials

The following are available online: HPLC chromatograms of phenolics of SBW extracts in Figures S1–S4, and HPLC chromatogram of monosaccharides of the granulated cork in Figure S5.
