# Supplementary material for: Valorization of Cork Using Subcritical Water
Source: Molecules. 2020 Oct 14;25(20):4695. doi: 10.3390/molecules25204695 (PMC7587334; doi:10.3390/molecules25204695)
Supplement: Supplementary file 1 [file molecules-25-04695-s001.pdf]

Supplementary Materials

Valorization of cork using subcritical water

Mónica Cunha *, Ana Lourenço, Susana Barreiros, Alexandre Paiva and Pedro Simões *

LAQV-REQUIMTE, Chemistry Department, Faculdade de Ciências e Tecnologia, Universidade Nova de Lisboa, 2829-516, Caparica, Portugal

List of Contents in Supplementary Materials

Figure S1. HPLC chromatogram of phenolics of SBW extract 1 (T < 50) obtained in the assay targeting 200 °C

Figure S2. HPLC chromatogram of phenolics of SBW extract 2 (T: 50 - 120) obtained in the assay targeting 200 °C

Figure S3. HPLC chromatogram of phenolics of SBW extract 3 (T: 120 - 200) obtained in the assay targeting 200 °C

Figure S4. HPLC chromatogram of phenolics of SBW extract 4 (T: 200) obtained in the assay targeting 200 °C

Figure S5. HPLC chromatogram of monosaccharides of the granulated cork

Additional Info : Peak(s) manually integrated

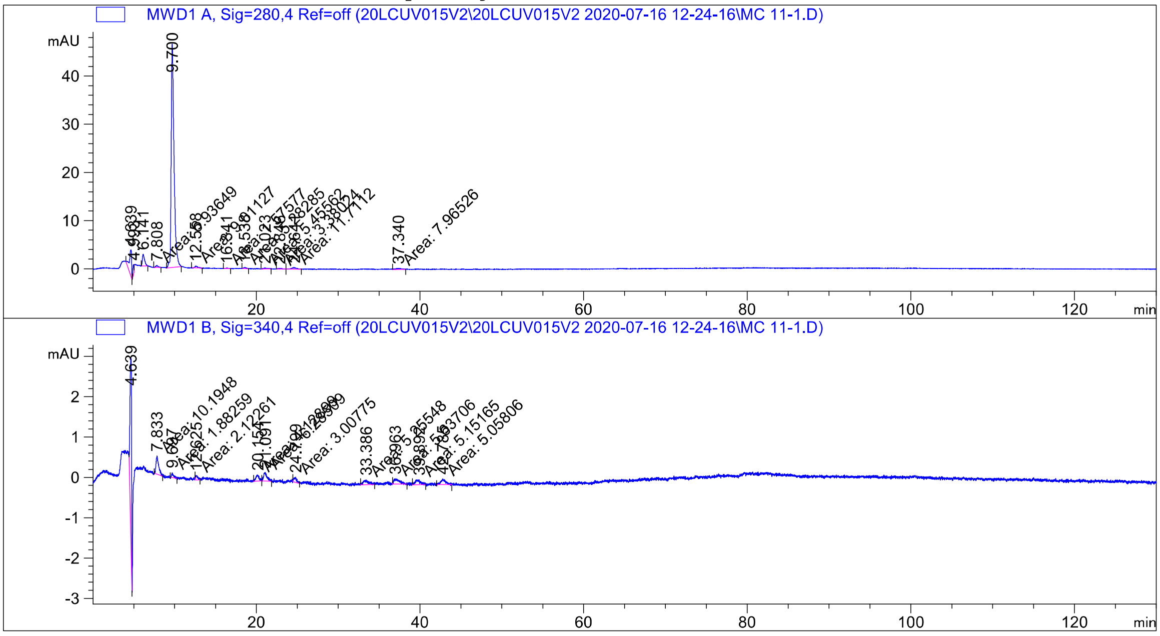

Figure S1. HPLC chromatogram of phenolics of SBW extract 1 (T < 50) obtained in the assay targeting 200 °C

Additional Info : Peak(s) manually integrated

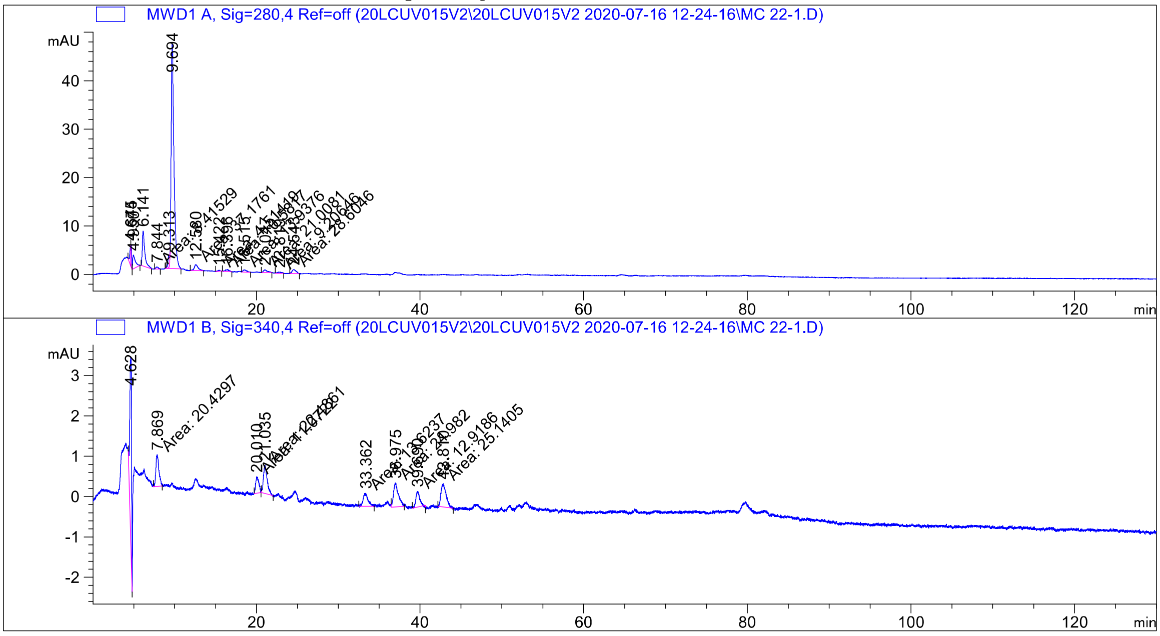

Figure S2. HPLC chromatogram of phenolics of SBW extract 2 (T: 50 - 120) obtained in the assay targeting 200 °C

Additional Info : Peak(s) manually integrated

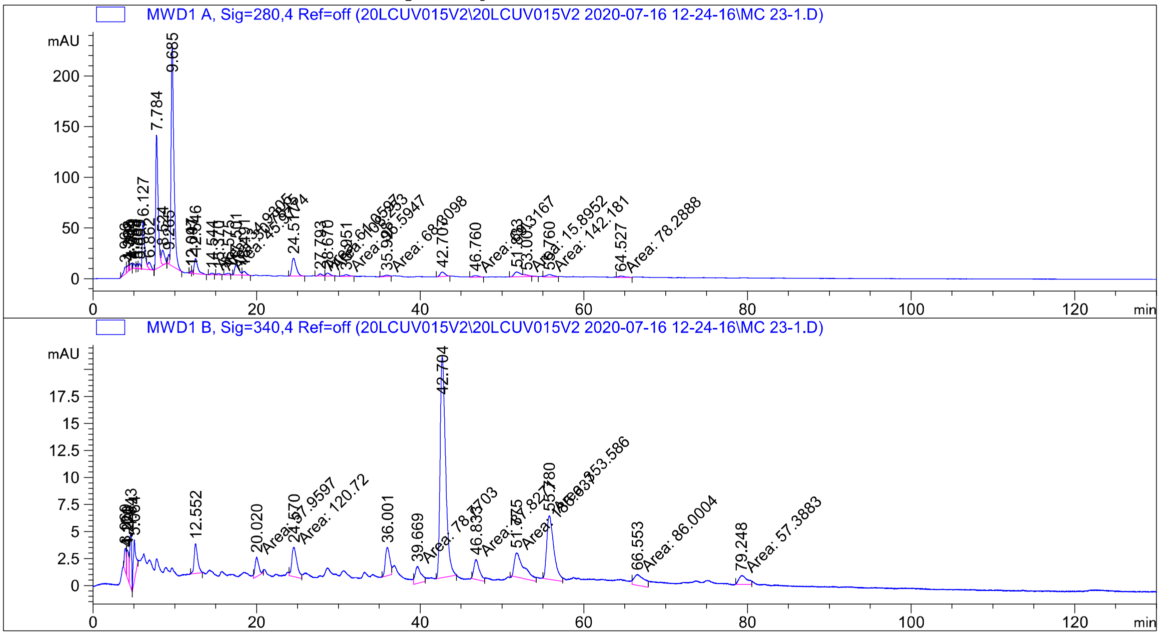

Figure S3. HPLC chromatogram of phenolics of SBW extract 3 (T: 120 - 200) obtained in the assay targeting 200 °C

Additional Info : Peak(s) manually integrated

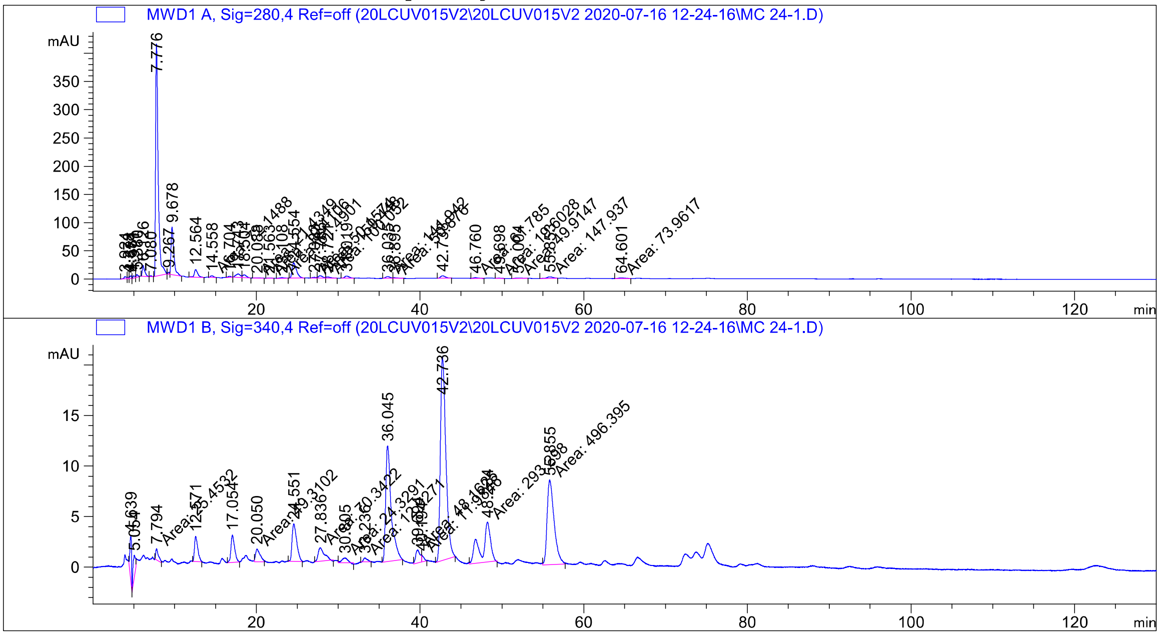

Figure S4. HPLC chromatogram of phenolics of SBW extract 4 (T: 200) obtained in the assay targeting 200 °C

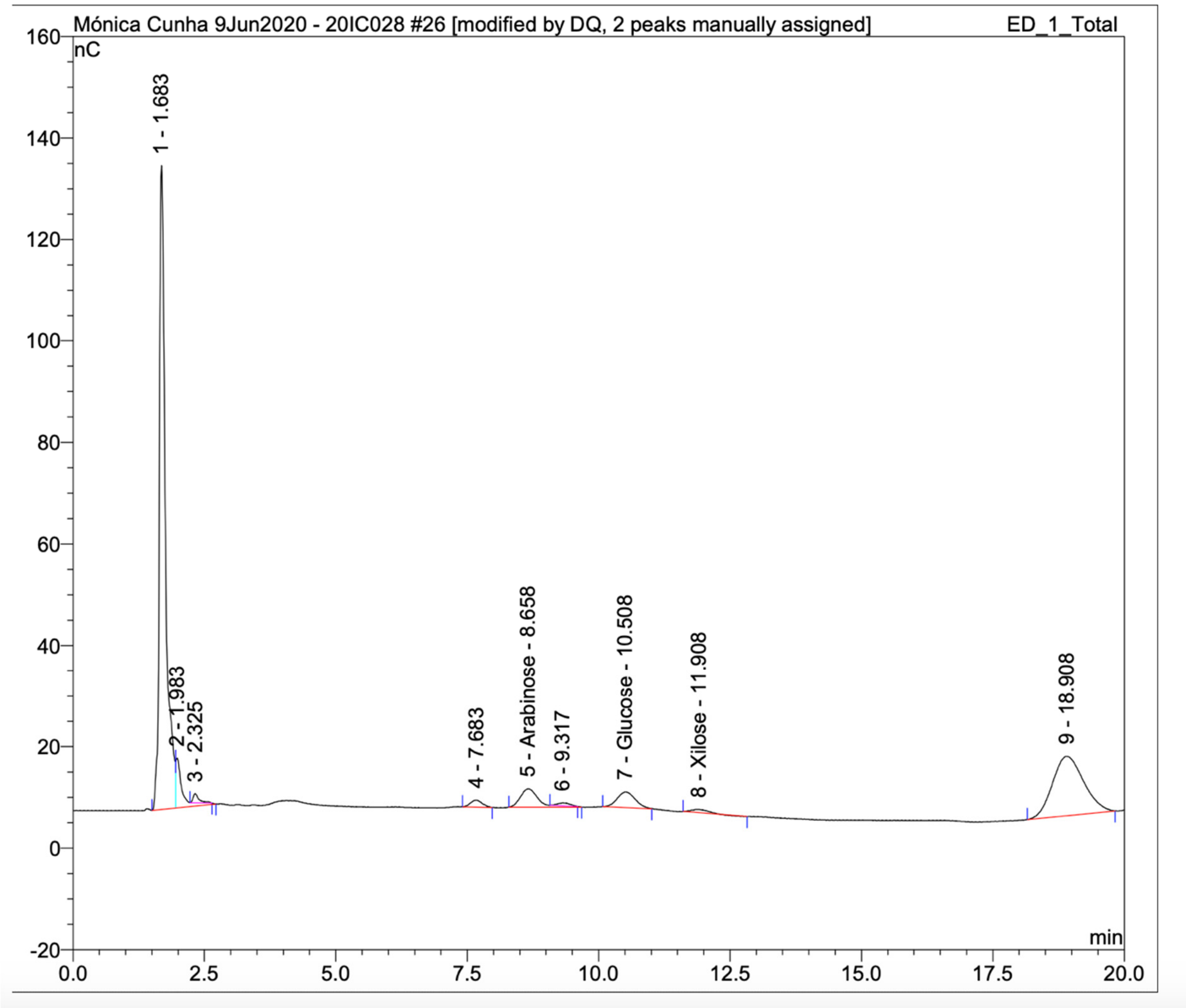

Figure S5. HPLC chromatogram of monosaccharides of the granulated cork
